# Fatores de Risco Cardiovascular em Cardiologistas Certificados pela Sociedade Brasileira de Cardiologia: Lições a serem Aprendidas

**DOI:** 10.36660/abc.20210153

**Published:** 2021-04-08

**Affiliations:** 1 Universidade Federal Fluminense Hospital Universitário Antônio Pedro (HUAP)/Ebserh Setor de Medicina Nuclear NiteróiRJ Brasil Setor de Medicina Nuclear - Hospital Universitário Antônio Pedro (HUAP)/Ebserh - Universidade Federal Fluminense (UFF), Niterói, RJ - Brasil.; 2 Hospital Pró-Cardíaco Serviço de Medicina Nuclear Rio de JaneiroRJ Brasil Serviço de Medicina Nuclear - Hospital Pró-Cardíaco, Rio de Janeiro, RJ - Brasil.; 3 Hospital Vitória Rio de JaneiroRJ Brasil Hospital Vitória, Rio de Janeiro, RJ – Brasil.

**Keywords:** Doença Arterial Coronária/mortalidade, Aterosclerose, Dislipidemias, Obesidade, Diabetes Mellitus, Fatores de Risco, Cardiologistas

As doenças cardiovasculares (DCV) continuam sendo a principal causa de morte no mundo. Fatores de risco como hipertensão arterial sistêmica (HAS), diabetes mellitus (DM), dislipidemia, obesidade, tabagismo e alcoolismo associados ao comportamento sedentário, privação de sono, estresse e histórico familiar favorecem a aterosclerose. A complexidade da fisiopatologia no processo de formação da aterosclerose e a variedade de fatores de risco para doença arterial têm impactos importantes na mortalidade.^1^^–^^3^ A HAS é um importante fator de risco, sendo o mais prevalente para DCV, e sua relação é decorrente de lesões vasculares que causam hiperplasia e hipertrofia da camada média do vaso. As doenças cardiovasculares são a principal causa de mortalidade e morbidade em pessoas com diabetes. Resulta da hiperglicemia e resistência à insulina, levando à inflamação crônica, estresse oxidativo e, em última instância, disfunção endotelial. A DM tem aumentado significativamente nos últimos anos, tornando-se uma das principais causas de mortalidade. A resistência à insulina promove dislipidemia, acelerando a aterosclerose em pacientes diabéticos.^4^^–^^7^ A prevalência da obesidade tem crescido muito nas últimas décadas. É uma entidade complexa, de grande impacto nas doenças cardiovasculares e importante no comprometimento dos fatores de risco (HAS, DM e dislipidemia). Há aumento da incidência de morte súbita entre pacientes obesos, geralmente por arritmias ventriculares frequentes e complexas. Tabagismo, ingestão de álcool e sedentarismo estão relacionados ao aumento do risco cardiovascular, afetando todas as fases da aterosclerose. A alimentação não saudável está relacionada a fatores que interferem na prevenção e controle das DCV, sendo de grande importância para a mortalidade precoce em todo o mundo. A adoção de hábitos saudáveis exige constante esforço pessoal e resiliência.^8^^–^^10^ A melhora no estilo de vida associada à alimentação saudável traz novas perspectivas para a prevenção cardiovascular. A atividade física tem efeitos positivos no metabolismo lipídico, glicose e pressão arterial e, combinada com novas classes de medicamentos para controlar os fatores de risco, resultou na diminuição de desfechos cardiovasculares. O controle do tabagismo é um importante instrumento de prevenção primária que, por meio da educação continuada nos últimos anos, tem levado a uma redução importante no número de dependentes.^8^^–^^11^

Em um novo estudo com cardiologistas, Teixeira e cols. encontraram menor prevalência de sedentarismo e tabagismo, mas maior prevalência de consumo de álcool. A prevalência de dislipidemia foi maior do que HAS e DM. Embora os especialistas tivessem maior conhecimento sobre a doença, não foi possível observar hábitos mais saudáveis do que o restante da população^12^^–^^14^ Em contrapartida, no Canadá, um estudo de coorte com 17.071 médicos em atividade e 5.306.038 membros da população em geral apontou que os médicos usavam menos serviços preventivos recomendados pelas diretrizes e tiveram taxas mais baixas de fatores de risco cardíaco. Após oito anos de acompanhamento, os médicos tiveram um risco substancialmente menor de desfechos adversos do que a população em geral.^15^ Esses resultados podem nos levar a especular sobre as possíveis causas dessas diferenças. O estilo de vida estressante e a jornada excessiva de trabalho atrelada à prática médica no Brasil podem ser responsáveis por parte desses achados.

A implementação de mudanças no estilo de vida, prevenção primária e secundária, aliada a terapia adequada e diagnóstico precoce, é fundamental para a redução das DCV. Apesar do nível de conhecimento dos profissionais de saúde, pouco se sabe sobre seus fatores de risco para DCV. No Brasil, os cardiologistas desempenham um papel importante na promoção da prevenção e tratamento de doenças cardiovasculares.^16^ Melhorias nas diretrizes clínicas trouxeram benefícios clínicos significativos para a prevenção de DCV. No entanto, existe uma necessidade importante não atendida: entender melhor os fatores que afetam os hábitos dos cardiologistas brasileiros e melhorar seus fatores de risco.^12^^–^^15^
