# Supplementary material for: Fatores de Risco Cardiovascular em Cardiologistas Certificados pela Sociedade Brasileira de Cardiologia: Lições a serem Aprendidas
Source: Arq Bras Cardiol. 2021 Apr 8;116(4):782–3. [Article in Portuguese] doi: 10.36660/abc.20210153 (PMC8121386; doi:10.36660/abc.20210153)
Supplement: Translation

# Translation: Cardiovascular Risk Factors in Cardiologists Certified by the Brazilian Society of Cardiology: Lessons to be Learned

**Affiliations:** 1 NiteróiRJ Brazil Setor de Medicina Nuclear - Hospital Universitário Antônio Pedro (HUAP)/Ebserh - Universidade Federal Fluminense (UFF), Niterói, RJ - Brazil.; 2 Rio de JaneiroRJ Brazil Serviço de Medicina Nuclear - Hospital Pró-Cardíaco, Rio de Janeiro, RJ - Brazil.; 3 Rio de JaneiroRJ Brazil Hospital Vitória, Rio de Janeiro, RJ – Brazil.

**Keywords:** Coronary Artery Disease/mortality, Atherosclerosis, Dyslipidemias, Obesity, Diabetes Mellitus, Risk Factors, Cardiologists

Cardiovascular diseases (CVD) remain the leading cause of death in the world. Risk factors such as systemic arterial hypertension (SAH), diabetes mellitus (DM), dyslipidemia, obesity, smoking and alcoholism associated with sedentary behavior, sleep deprivation, stress and family history favor atherosclerosis. The complexity of pathophysiology in the process of atherosclerosis formation and the variety of risk factors for artery disease have important impacts on mortality.^1^^–^^3^ SAH is an important risk factor, being the most prevalent for CVD and its relationship is the result of vascular lesions that cause hyperplasia and hypertrophy of the middle layer of the vessel. Cardiovascular disease is the main cause of mortality and morbidity in people with diabetes. It is the result of hyperglycemia and insulin resistance, leading to chronic inflammation, oxidative stress and, ultimately, endothelial dysfunction. DM has increased significantly in recent years, making it one of the main causes of mortality. Insulin resistance promotes dyslipidemia, accelerating atherosclerosis in diabetic patients.^4^^–^^7^ Obesity prevalence has grown greatly in recent decades. It is a complex condition that has a great impact on cardiovascular diseases and plays an important role in affecting risk factors (SAH, DM and dyslipidemia). Sudden death is increased in obese patients, usually by frequent and complex ventricular arrhythmias.^11^ Smoking, alcohol intake and sedentary lifestyle are related to increased cardiovascular risk affecting all phases of atherosclerosis.^9^^,^^10^ Unhealthy eating is related to factors that interfere with the prevention and control of CVD, being of great importance for early mortality around the world. The adoption of healthy habits requires constant personal efforts and resilience.^8^^–^^10^ Improvement in lifestyle associated with healthy eating provides new perspectives on cardiovascular prevention. Physical activity has positive effects on lipid metabolism, glucose, and blood pressure combined with new classes of drugs to control risk factors has resulted in lower cardiovascular outcomes. Tobacco control is an important instrument in primary prevention which, through continuing education in recent years, has seen an important reduction in the number of people dependent on smoking.^8^^–^^11^

In a new study with cardiologists, Teixeira et al. found lower prevalence of sedentary lifestyle and smoking, but a higher prevalence of alcohol consumption. The prevalence of dyslipidemia was higher than SAH and DM. Although specialists had greater knowledge about the disease, it was not possible to observe healthier habits than the rest of the population.^12^^–^^14^ In contrast, in Canada, a cohort study of 17,071 practicing physicians and 5,306,038 members of the general population found that physicians used fewer guideline-recommended preventive services and had lower rates of cardiac risk factors. After 8 years’ follow-up, physicians had a substantially lower risk of adverse outcomes than the general population.^15^ These results could lead us to speculate about the possible causes for these differences. A stressful lifestyle and excessive working hours linked to medical practice in Brazil could be responsible for part of these findings.

The implementation of lifestyle changes, primary and secondary prevention combined with appropriate therapy and early diagnosis is fundamental to reduce CVD. Despite the level of knowledge of health professionals, little is known about their risk factors for CVD. In Brazil, cardiologists play an important role in promoting prevention and treatment of cardiovascular diseases.^16^ Improvements in clinical guidelines have brought significant clinical benefits to the prevention of CVD. However, there is an important unmet need: to better understand the factors that impact Brazilian cardiologists’ habits, and to improve their risk factors.^12^^–^^15^
